# Person-centred and efficient care delivery for high-need, high-cost patients: primary care professionals’ experiences

**DOI:** 10.1186/s12875-020-01172-3

**Published:** 2020-06-11

**Authors:** Rowan G. M. Smeets, Mariëlle E. A. L. Kroese, Dirk Ruwaard, Niels Hameleers, Arianne M. J. Elissen

**Affiliations:** Department of Health Services Research, Care and Public Health Research Institute (CAPHRI), Faculty of Health, Medicine and Life Sciences, Maastricht University, Maastricht, The Netherlands

**Keywords:** Primary health care, Qualitative research, Delivery of health care, integrated

## Abstract

**Background:**

High-need, high-cost (HNHC) patients, who typically have complex and long-term care demands, contribute considerably to the high work pressure of primary care professionals (PCPs). To improve patient as well as provider experiences, it is crucial to take into account the PCPs’ perspective in designing health care strategies for HNHC patients. Therefore, this study aimed to create insight into PCPs’ experienced barriers and possible solutions with regards to person-centred, efficient care delivery to HNHC patients.

**Methods:**

We conducted a qualitative study using focus group interviews with PCPs at a Dutch primary care group. A semi-structured interview guide was developed for the interviews. Qualitative content analysis was employed deductively by means of a categorisation matrix. The matrix was based on the components retrieved from the SELFIE framework for integrated care for multi-morbidity.

**Results:**

Forty-two PCPs participated in five focus group interviews. Discussed barriers and solutions were related to the core of the SELFIE framework (i.e. the individual and environment), and particularly four of the six health system components in the framework: service delivery, leadership & governance, workforce, and technologies & medical products. Many discussed barriers revolved around the complex biopsychosocial needs of HNHC patients: PCPs reported a lack of time (service delivery), insufficiently skilled PCPs (workforce), and inefficient patient information retrieval and sharing (technologies & medical products) as barriers to adequately meet the biopsychosocial needs of HNHC patients.

**Conclusions:**

This qualitative study suggests that primary care is currently insufficiently equipped to accommodate the complex biopsychosocial needs of HNHC patients. Therefore, it is firstly important to strengthen primary care internally, taking into account the experienced lack of time, the insufficient number of equipped PCPs and lack of inter-professional information retrieval and sharing. Secondly, PCPs should be supported in cooperating and communicating more efficiently with health services outside primary care to adequately deliver person-centred, efficient care. As a prerequisite, it is crucial to direct policy efforts at the design of a strong system of social and community services. In terms of future research, it is important to assess the feasibility and effects of re-designing primary care based on the provided recommendations.

## Background

In 2014, Bodenheimer and Sinsky [1] proposed expanding the Triple Aim to a Quadruple Aim. Specifically, the authors added the improvement of provider experience to the already existing aims of improving patient experience, improving population health, and reducing per capita costs [2]. The need for increased attention for provider experience was underlined by studies showing the growing prevalence of burnout among healthcare professionals, in particular among primary care professionals (PCPs) [1, 3]. On the individual provider level, burnout is correlated with the prevalence of severe disorders, like depression and alcohol abuse [1, 4]. Moreover, some studies showed that provider burnout is negatively associated with quality and safety of patient care, and may increase health care costs [4, 5].

Previous studies suggest that many factors contribute to the rising work pressure in primary care. For instance, PCPs reported a changing work environment with large administrative tasks and non-face-to-face activities [1, 6–8]. From a wider, system perspective, an important contributing factor is the growing population of patients with chronic conditions and multimorbidity. The increase in number of chronically ill treated in primary care is not only a result of socio-demographic transitions, but also a (policy) tendency to transfer care tasks from hospital and community to primary care settings [9, 10]. As a result, primary care is faced with increased work pressure, alongside growing complexity of care demands which used to be dealt with in more specialised settings.

As an opportunity to improve provider experience, it is important to move towards more person-centred, efficient care delivery for chronically ill who have a disproportionately high care use. These patients are referred to as ‘high-need, high-cost’ (HNHC) patients [11–13]. Many studies have aimed to better understand the characteristics and needs of the HNHC patient population, in order to inform more high-quality care and lower costs [11–13]. Recent studies showed that the HNHC patient population cannot be captured only in a stereotype of clinical and biomedical complexity (e.g. multimorbidity, high prevalence of mental illness) and higher age [12, 14]. Rather, the HNHC patient population was found to be heterogeneous in terms of biopsychosocial characteristics (e.g. type of chronic conditions, age, and source of income) [11, 12, 14, 15].

While there is increasing insight into the characteristics and needs of the HNHC patient population, only a limited number of studies has addressed the experiences of PCPs with regards to care delivery to this population [16–19]. Taking into account the experiences of PCPs is crucial to create more person-centred, efficient care for the HNHC chronically ill patient population in primary care and, in so doing, to support efforts to move towards the Quadruple Aim [1]. Therefore, this study aimed to create insight into the experienced barriers and possible solutions with regards to person-centred, efficient care delivery to the HNHC patient population.

## Methods

### Setting

The present study was conducted at a primary care group in a northern, rural region of the Netherlands, covering 135 general practices and approximately 490,000 patients. In the Netherlands, chronically ill are mainly treated in a primary care setting. Many care tasks for chronically ill are currently transferred to practice nurses, with the general practitioner (GP) having a coordinating role. Practice nurses were first introduced in Dutch primary care in 2000, initially to provide care to patients with somatic chronic conditions (i.e. ‘somatic practice nurse’), such as diabetes [20]. In 2008, a second type of practice nurse, the ‘mental health practice nurse’ was introduced to deal with the increasing demands for mental health care in general practice [20–23].

### HNHC patient population

We defined the HNHC patient population in the participating primary care group as all chronically ill patients, who: (1) belonged to the top-10% of care utilisers; or (2) had multimorbidity in combination with an above-average care utilisation. In a previous study using this definition, we found that the HNHC patient population (using data from 63 practices and 12,602 HNHC patients) consists of four subgroups with distinct biopsychosocial profiles [15]. Although these profiles are multidimensional, they can be characterised as: (1) older adults living with partner; (2) older adults living alone; (3) middle-aged, employed adults with family; and (4) middle-aged adults with social welfare dependency [15].

The biopsychosocial heterogeneity of the HNHC patient population was illustrated by case descriptions in this study. These case descriptions were discussed at the beginning of each focus group. Four case descriptions were established, each describing one ‘typical’ patient of the four previously identified HNHC patient subgroups with distinct biopsychosocial characteristics. This means that the case descriptions included the following information of a ‘typical’ patient: patients’ mean age, their most prevalent household position, source of income, and (top-5 prevalent) chronic conditions, and their health care use outside primary care (based on mean health care costs). To illustrate this, we developed the following case description for the subgroup of ‘middle-aged adults with social welfare dependency’: *Ms. Smith is 52 years old and living alone for some time now. Due to severe mood disorders, she is dependent on sickness benefits. Besides the mood disorders, she has been suffering from asthma since her youth. For a couple of years, she receives care from a specialised mental health professional, alongside the care she receives from the GP*.

### Focus group participants

In this qualitative study, focus group interviewing with PCPs was employed to collect a variety of experiences from interactive discussion [24–26]. In order to interview a relatively large number of PCPs, the method of convenient sampling was used. PCPs of the first two focus groups were gathered via a regional meeting for (somatic) practice nurses; PCPs of the following three focus groups were gathered via a primary care conference that was attended by various types of PCPs (i.e. GPs, practice nurses, doctor’s assistants). Before the interviews, PCPs were given assurances about the confidentiality of their contribution and were asked for verbal informed consent to participate in the study and audiotape their responses.

### Focus group interviews

Five focus group interviews were organised: two interviews lasted approximately 90 min, the remaining three lasted approximately 60 min. The interviews were organised at the location of the regional meeting and the conference (where PCPs were sampled). The focus group interviews were conducted by one author (RS or MK) and observed by another author (AE or NH) or the (somatic) practice nurse of the primary care group. The observers wrote down keywords from the interview on a flip-over, and complemented the researcher who conducted the interviews with follow-up questions. The interviews were audio-taped.

A semi-structured interview guide was developed for conducting the interviews. The guide was pre-tested with a (somatic) practice nurse to check the clarity and validity of the guide. The first theme included in the guide pertained to the experienced barriers with regards to person-centred, efficient care delivery to the HNHC patient population. To initiate the discussion on the experienced barriers, the PCPs were asked to fill in an assignment on the top-3 most important barriers. The second theme included in the guide pertained to the experienced possible solutions with regards to person-centred, efficient care delivery to the HNHC patient population.

### Data analysis

Various theoretical models and frameworks for integrated care to patients with multimorbidity were introduced over the years [27, 28]. In the current study, we selected the ‘Sustainable intEgrated chronic care modeLs for multi-morbidity: delivery, FInancing, and performance (SELFIE)’ framework to deductively analyse our focus group data, as it specifies important concepts for integrated care in a comprehensive way [27]. Furthermore, the application of the SELFIE framework can add to the systematic categorization and comparison of interview data. The SELFIE framework has categorised relevant concepts for integrated care according to six (adapted WHO health systems) components, each having three different levels (micro, meso, macro): service delivery, leadership & governance, workforce, financing, technologies & medical products, and information & research. In addition, a holistic understanding of the individual with multi-morbidity and his/her environment is positioned centrally in the framework.

To analyse the data, qualitative content analysis was applied using a three-stage process: data preparation, organisation (i.e. analysis), and reporting [29]. In the preparation stage, the interviews were transcribed verbatim [29, 30]. After repeatedly reading the interview transcripts in order to get acquainted with the data, a structured categorisation matrix was developed based on the SELFIE framework for coding purposes [27]. The matrix enabled categorization of the interview data according to 20 codes, derived from the SELFIE framework (see Table 1): one for the individual HNHC patient, one for his/her environment, and one for each level within the six components of the SELFIE framework. A code book with explanations and examples of the components from the SELFIE framework was developed to ensure a valid coding process. In the organisation stage, the data were stepwise organised according to the codes included in the matrix. This process supported the description of the data and identification of patterns within the data. Researchers RS and AE discussed the validity and consistency of the applied codes: disagreements were resolved by discussion. To facilitate the organisation stage, The Qualitative Data Analysis & Research Software ATLAS.ti (version 8.0) was used.

**Table 1:**
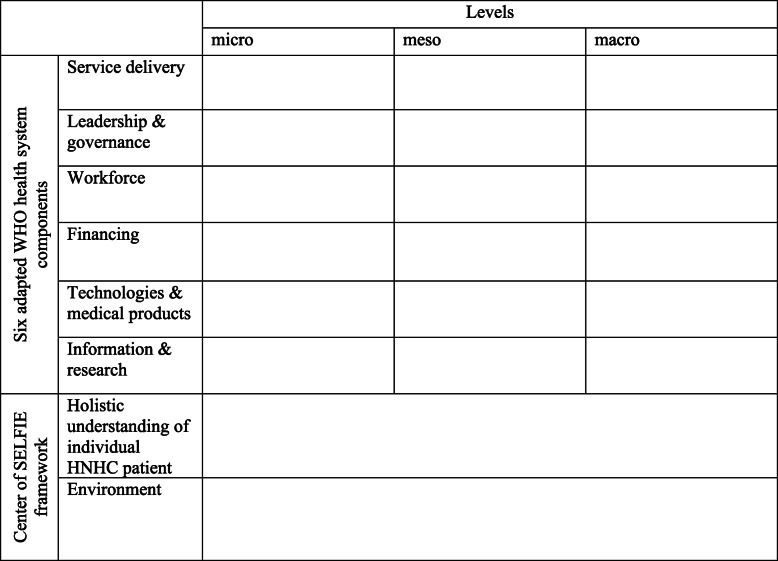
Categorisation matrix, derived from the SELFIE framework [27]

## Results

Forty-two PCPs participated in five focus group interviews (see Table 2 for background characteristics). The experienced barriers and possible solutions with regards to person-centred, efficient care delivery to the HNHC patient population are described below by the SELFIE framework, starting with the centre of the framework (individual and environment), followed by the six components for integrated care [27]. The barriers as well as the solutions are described from micro to macro level.

**Table 2:** Background characteristics of PCPs (*n* = 42) who participated in focus group interviews

Characteristic	Total (n = 42)	Focus group 1 (n = 7)	Focus group 2 (*n* = 7)	Focus group 3 (*n* = 6)	Focus group 4 (*n* = 8)	Focus group 5 (*n* = 14)
	n (%)					
Sex						
Male	11 (26.2%)	0	0	1 (16.7%)	3 (37.5%)	7 (50%)
Female	30 (71.4%)	7 (100%)	7 (100%)	5 (83.3%)	5 (62.5%)	6 (42.9%)
Missing	1 (2.4%)	0	0	0	0	1 (7.1%)
Age, mean (SD)	46.7 (10.7)	41.9 (9.3)	54.7 (3.7)	46.2 (15.3)	45.0 (11.9)	46.9 (9.6)
Missing, n (%)	3 (7.1%)	0	1 (14.3%)	0	0	2 (14.3%)
Primary care profession						
Somatic practice nurse	19 (45.2%)	7 (100%)	7 (100%)	1 (16.7%)	2 (25%)	2 (14.3%)
GP	13 (31.0%)	0	0	3 (50%)	3 (37.5%)	7 (50%)
Other^a^	9 (21.4%)	0	0	2 (33.3%)	3 (37.5%)	4 (28.6%)
Missing	1 (2.4%)	0	0	0	0	1 (7.1%)
GP practice type						
General practice not part of multi-disciplinary health centre	26 (61.9%)	7 (100%)	5 (71.4%)	4 (66.7%)	4 (50%)	6 (42.9%)
General practice part of multi-disciplinary health centre	10 (23.8%)	0	2 (28.6%)	1 (16.7%)	3 (37.5%)	4 (28.6%)
Not applicable	4 (9.5%)	0	0	1 (16.7%)	0	3 (21.4%)
Missing	2 (4.8%)	0	0	0	1 (12.5%)	1 (7.1%)
Working experience in general practice (years), mean (SD)	14.8 (10.4)	15.9 (13.5)	12.3 (16.2)	9.1 (7.4)	10.1 (7.3)	21.2 (10.5)
Not applicable, n (%)	4 (9.5%)	1 (14.3%)	0	1 (16.7%)	0	2 (14.3%)
Missing, n (%)	3 (7.1%)	0	1 (14.3%)	0	1 (12.5%)	1 (7.1%)

^a^Other professions included doctor’s assistant (n = 4), coordinator elderly care at care group (n = 1), programme manager pulmonary medicine (n = 1), policy advisor (n = 1), retired GP (n = 1), manager of care group (n = 1)

### Individual HNHC patient

PCPs characterised the HNHC patient by a high burden of mental (e.g. dementia) and psychosocial problems (e.g. loneliness in older patients). In addition, it was reported that older HNHC patients who are living alone may have a tendency to avoid care. Due to these mental and psychosocial problems, PCPs reported that patients experience increased difficulty to efficiently manage their (physical) chronic conditions and improve their health:

*“If you [as a patient] had a good weekend, then there is less urgency to visit the GP on Monday morning. [ …*] *I sometimes think: “What does this patient want?” It is just that this patient has nothing else to do. […*] *Of those 35% [of patient population] who visits the GP every single day, 80% has to deal with psychosocial problems.”* (FG3).

### Environment

PCPs reported a lack of sufficient informal care provision and a limited social network of some HNHC patients, also due to insufficient possibilities for PCPs to find volunteers. In particular in more urban (compared to rural) areas, (older-aged) HNHC patients may experience challenges in maintaining a supportive social network:

*“I see a difference between the villages and the more urban population. I live in a village with strongly connected communities where people look after each other. I had a neighbour who took care of everything herself until she was in her nineties. [ …*] *Her kids live far away, but she could take care of herself because of us [the community].”* (FG3).

Furthermore, while a patient’s partner can be supportive towards the patient, a partner can also have a more negative influence on the patient which can lead to increased care demands (e.g. a partner who is highly dominant). In addition, it was reported that some HNHC patients, in particular middle-aged employed patients, do not have enough time (due to a high burden of work and providing informal care) to visit the GP or do not have enough money to take the required medical examinations. Consequently, it can be challenging to have a clear overview over the patient’s health situation. Also, PCPs mentioned that it is difficult to discuss poverty with patients.

### Service delivery

It was mentioned that a lack of time is often experienced during consultations to approach patients in a holistic way and address psychosocial problems:

*“If you take a look at what the consultations are about, then I sometimes wonder whether it is about the physical problems, or about people who want to share their story. There is always something alongside [the physical problem] that leads to the mental problem. [ …*] *As a result, it is difficult to set goals and it is also much more difficult to achieve those goals.”* (FG1).

Furthermore, PCPs mentioned insufficient time is reserved for acute care demands (e.g. patients with deteriorated blood sugar control) which leads to increased workload. PCPs also mentioned that they spend increased time on prevention and pro-active care (e.g. screening for co-morbidities). In addition, care delivery is complicated by common treatment interaction issues (e.g. polypharmacy) in HNHC patients. PCPs reported that patients have, over the years, perceived primary care as increasingly accessible care which increases their use. Mental health care is nonetheless perceived less accessible; moreover, there is a certain extent of stigma around mental health care use in the Netherlands. As a result, patients prefer to visit the GP or somatic practice nurse, even though their complex needs require more specialised care.

In terms of possible solutions, PCPs suggested to introduce expanded consultations to enable a more holistic approach:

*“I have scheduled five [instead of six] consultations in one hour, which means that [ …*] *my consultations are substantially different. Which means that other things are addressed, which implies that I am able to solve more in just one consultation.”* (FG5).

Also, PCPs discussed the importance of involving the informal caregiver in order to discuss the health situation of the patient (in particular for older HNHC patients who have an informal caregiver). At the same time, PCPs report challenges when an informal caregiver has a different opinion on the health status of the patient than the patient has. PCPs furthermore suggested to better integrate disease programmes and integrate care services into accessible multidisciplinary health centres as HNHC patients have diverse and complex needs:

*“It would be good if, like it used to be, there would be one centre in one community with a GP, with a social worker [ …*] *where all disciplines are located. They [the care professionals] are familiar with the community and people can easily come by.”* (FG4).

### Leadership & governance

PCPs discussed policy efforts that stimulate task referral from settings outside primary care (like residential elderly care or the community setting) to primary care. This generally increases work pressure and complicates care delivery in primary care:

*“These are the [older-aged] people who used to be institutionalised in a nursing home and who could participate with activities like drinking coffee and knitting, who are now just living alone at home.[...] These are the people who say: “Well, I will visit the GP to check if everything is okay”.”* (FG5).

Moreover, policy efforts focusing on the introduction of free market principles in health care were mentioned, which have led to an unstable market for home care organisations in the Netherlands, with many mergers and bankruptcies. Consequently, PCPs mentioned that it is challenging to keep an overview of and communicate adequately with home care organisations.

With regards to solutions, PCPs noted the importance of shared decision-making and individualised care planning in order to improve the health of patients. For instance, PCPs suggested to set small and achievable goals for patients and discuss the financial feasibility of examinations with the patient (particularly in case the patient has to deal with poverty).

### Workforce

It was reported that communication between different professionals within and beyond the boundaries of primary care is sometimes inadequate. This can lead to inefficiencies in care delivery (e.g. inadequate information sharing). In addition, PCPs miss an overview of the different involved professionals in care delivery. Due to patients’ complex needs and the variety of involved care professionals, PCPs moreover experience it as increasingly challenging to function as the ‘named coordinator’:

*“There are people [HNHC patients] who see many different medical specialists and then [ …*] *it can be very complex, but you [as a PCP] are the coordinator who should maintain the overview.”* (FG1).

Due to the increasingly complex and psychosocial demands of HNHC patients, PCPs reported that their traditional role gets expanded. Also, PCPs indicated it as challenging to offer sufficient support to the informal caregiver (of older HNHC patients) during consultations. PCPs furthermore reported that the volume and diversity of the primary care workforce does not always adequately accommodate the growing work pressure. For instance, an insufficient number of PCPs is available in order to be able to expand the consultation time per HNHC patient. In addition, some PCPs discussed that their professional education spend limited attention to psychosocial problems like loneliness.

Related to solutions, PCPs mentioned that cooperation with various disciplines (in multi-disciplinary meetings) is crucial for integrated, high-quality care to HNHC patients. Multi-disciplinary meetings are thought to unite different professional perspectives and enable efficient task division:

*“I think our practice is very well organised with regards to multi-disciplinary meetings with different disciplines. You take a look at the patient’s problems from different professional perspectives and then, yes, you can come up with a solution I think.”* (FG2).

Furthermore, many PCPs suggested the introduction of new, expanded roles or a more efficient task division to deal with the increasing complex patient demands and associated workload:

*“Sometimes I think that someone like this [patient receiving social welfare benefits] should just have a coach, who helps to get their life together. [ …*] *not only financially but also to help in making the right decisions, for example finding a job in society.”* (FG1).

### Financing

PCPs discussed that some important programmes (e.g. social event for older adults) and care services (e.g. physiotherapy) are not sufficiently financially covered and reimbursed generously enough. This implies that these types of services which are required for HNHC patients, due to their complex biopsychosocial problems, may not always be (financially) accessible.

### Technologies & medical products

PCPs reported to experience a high burden of (growing) administrative tasks, especially when their general practice is connected to a pharmacy. This results in less available time for patients during consultations. Also, psychosocial patient information is largely lacking in electronic health records (EHRs), although this can facilitate a holistic approach:

*“It would be very good to have a bit of background information of each patient, like where the patient lives, the household situation, who is the informal caregiver. But it is difficult where to register this information [ …*] *as you cannot remember all this information. [ …*] *This is a matter of ICT. That is the main barrier.”* (FG2).

Some PCPs, on the other hand, commented that the registration of psychosocial patient information may increase the work load. Moreover, PCPs reported that EHRs do not facilitate optimal and most efficient registration or retrieval of relevant patient data. For instance, information for the same patient needs to be registered in different screens. There is also a lack of adequate shared information systems, which leads to inefficiencies and poor inter-professional communication:

*“We would like those [ICT] systems to be connected to each other. [ …*] *The community nurse works with her own [ICT] system and the GPs work with the EHR. If we could connect those to each other. It is just actually three systems to be connected and then it covers it all.”* (FG2).

### Information & research

PCPs reported to be sometimes uncertain about the data they are allowed to register, for example related to the patient’s work-related health issues. In terms of solutions, PCPs discussed the potential added value of stratifying their patient population into risk profiles. This stratification can be used to determine required care and spend more attention to specific patients with high needs.

## Discussion

### Summary

PCPs experience a comprehensive set of barriers with regards to the delivery of person-centred, efficient care to HNHC patients in primary care. Main barriers and solutions were related to the core of the SELFIE framework (i.e. the individual and his/her environment), as well as to (in particular) four of the six health system components of the framework: service delivery, leadership & governance, workforce, and technologies & medical products. Only a limited number of discussed barriers and solutions were directly related to the components of financing, and information & research.

### Strengths and limitations

A strength of this study was that not only experienced barriers but also possible solutions were discussed during interviews. In addition, a relatively large number of PCPs with different professional backgrounds, i.e. GPs and somatic practice nurses, were interviewed. However, another important PCP with regards to care delivery to HNHC patients, the mental health practice nurse [20, 23], was missing in the sample as a result of convenience sampling. After all, many HNHC patients have to deal with mental and psychosocial problems which underlines the important role of the mental health practice nurse in their care delivery [15].

### Comparison with existing literature

In relation to the core of the SELFIE framework (i.e. individual patient and environment), the current study indicates that HNHC patients generally have to deal with complex biopsychosocial health problems. Often, HNHC patients’ ability to deal with these complex problems is further challenged by their environment. For instance, older-aged HNHC patients may have a limited social network, which can lead to psychosocial issues like loneliness. On the other hand, employed HNHC patients may experience challenges in prioritising their health, as they have to balance, for example, work with informal caregiving to family members. The biopsychosocial complexity of HNHC patients as well as their various individual and environmental characteristics that are typically present in HNHC patients is also supported by previous studies [13, 14, 17, 18]. Also, it was previously found that these characteristics can negatively affect a patient’s ability to manage his/her health adequately, for instance by limiting the ability to understand and adequately follow treatment advice [19].

The barriers experienced by PCPs are related to different, but in particular four, health system components. This suggests a need for investment in a comprehensive set of interacting health system components to improve care for the HNHC patient population. In the majority of these components, i.e. service delivery, workforce, and technologies & medical products, experienced barriers relate to a micro or meso level. These can be summarised as a lack of time to address psychosocial problems, an insufficient number of PCPs skilled to address the complex, multidimensional needs of HNHC patients, and a lack of efficient inter-professional patient information retrieval and sharing. Only in one of the four most discussed components, i.e. leadership & governance, experienced barriers mainly relate to a macro level: policy efforts that (sometimes unintentionally) stimulate the transfer of complex care tasks to primary care. This may imply that PCPs predominantly experience barriers in the individual interaction with patients and on an organisational practice level. The SELFIE framework nonetheless underlines that integrated care requires alignment of macro level policies and regulations with the lower levels [27]. Only a limited number of barriers relate to the components of financing, and information & research. However, it can be argued that many of the discussed barriers are in fact related to or influenced by the underlying payment system. For instance, in order to stimulate more efficient cooperation and information sharing between disciplines, it is crucial to introduce payments systems that incentivise more collaboration. Also, reimbursement structures should allow the expansion of consultation time in case of complex needs [27]. Smith, O’Kelly [17] have previously argued that although more time for patients with multimorbidity is experienced as a crucial solution, the broad set of additional solutions in particular supports the design of complex, comprehensive interventions.

It should be noted that the current study was conducted in a predominantly rural region in the Netherlands. However, it is expected that the reported barriers are also largely valid for practices located in other, (more) urban regions. After all, the Netherlands is a small, densely populated country, with limited regional differences between general practices, for instance related to PCPs’ workload or care coordination [31–35]. The latter has significantly improved in both rural and urban areas due to the introduction of regional primary care groups, currently covering almost the entire country [32–34]. Care groups support practices in offering integrated chronic care under a bundled payment system [32–34]. In addition, several studies found no significant differences between rural and urban regions in terms of PCPs’ workload, which is relatively high in all primary care regions [31, 35].

### Implications for practice and research

Firstly, PCPs should be enabled to spend more attention to the biopsychosocial complexities of HNHC patients, including the individual and environmental characteristics interacting with these complexities. This calls for re-organising primary care internally: taking into account the experienced lack of time, the insufficient number of equipped PCPs and lack of inter-professional information retrieval and sharing is crucial. Secondly, PCPs should be supported in cooperating and communicating more efficiently with health services outside primary care to adequately deliver person-centred, efficient care.

In order to strengthen primary care and stimulate adequate cooperation, a starting point may be to design expanded consultations for HNHC patients which specifically aim at increasing insight into biopsychosocial health issues of HNHC patients. Ideally, these consultations are led by PCPs who are specifically trained in the assessment and coordination of complex biopsychosocial needs. To efficiently assess the biopsychosocial complexities, it may be helpful to use a biopsychosocial assessment tool. An example of such a tool is the Patient Centered Assessment Method, which was designed for “assessing patient complexity in ways that are sensitive to the biopsychosocial dimensions of health” [36]. Informed by the assessment of biopsychosocial complexities, PCPs can determine the type and degree of inter-professional cooperation and communication that is required. A prerequisite for adequate cooperation is to have sufficient insight into involved disciplines and the network of available health services outside primary care. Furthermore, to enhance primary care and stimulate cooperation, several policy efforts need to be aligned. Amongst others, it is important to direct policy efforts at the design of a strong system of social and community services.

## Conclusions

The present qualitative study suggests that the current system of care delivery within primary care is insufficiently equipped to accommodate the complex biopsychosocial needs of HNHC patients. To overcome those barriers and work towards the Quadruple Aim, comprehensive strategies are needed that not only strengthen primary care internally, but also support more adequate inter-professional cooperation and communication.

## Data Availability

The qualitative data collected and analysed during the current study may be available from the corresponding author on reasonable request.

## Abbreviations

GP: General practitioner
HNHC: High-need, high-cost
PCP: Primary care professional
SELFIE: Sustainable intEgrated chronic care modeLs for multi-morbidity: delivery, FInancing, and performance
